# Metabolomics Reveals Distinctive Metabolic Profiles and Marker Compounds of Camellia (*Camellia sinensis* L.) Bee Pollen

**DOI:** 10.3390/foods12142661

**Published:** 2023-07-11

**Authors:** Dandan Qi, Meiling Lu, Jianke Li, Chuan Ma

**Affiliations:** 1 State Key Laboratory of Resource Insects, Institute of Apicultural Research, Chinese Academy of Agricultural Sciences, No. 2 Yuanmingyuan West Road, Haidian District, Beijing 100193, China; qidandan07@126.com (D.Q.); apislijk@126.com (J.L.); 2 Tea Research Institute, Shangdong Academy of Agricultural Sciences, Jinan 250000, China; 3 Agilent Technologies (China) Co., Ltd., Beijing 100102, China; mei-ling.lu@agilent.com

**Keywords:** camellia bee pollen, L-theanine, epicatechin gallate, marker compound, botanical origin

## Abstract

Camellia bee pollen (CBP) is a major kind of bee product which is collected by honeybees from tea tree (*Camellia sinensis* L.) flowers and agglutinated into pellets via oral secretion. Due to its special healthcare value, the authenticity of its botanical origin is of great interest. This study aimed at distinguishing CBP from other bee pollen, including rose, apricot, lotus, rape, and wuweizi bee pollen, based on a non-targeted metabolomics approach using ultra-high performance liquid chromatography–mass spectrometry. Among the bee pollen groups, 54 differential compounds were identified, including flavonol glycosides and flavone glycosides, catechins, amino acids, and organic acids. A clear separation between CBP and all other samples was observed in the score plots of the principal component analysis, indicating distinctive metabolic profiles of CBP. Notably, L-theanine (864.83–2204.26 mg/kg) and epicatechin gallate (94.08–401.82 mg/kg) were identified exclusively in all CBP and were proposed as marker compounds of CBP. Our study unravels the distinctive metabolic profiles of CBP and provides specific and quantified metabolite indicators for the assessment of authentic CBP.

## 1. Introduction

Bee pollen is collected from plant flowers and agglutinated into pellets by honeybees via oral secretion. As an indispensable nutrient source for honeybee development, bee pollen is rich in carbohydrates, proteins, amino acids, polyphenols, lipids, minerals, and vitamins [1]. Its chemical composition varies considerably according to its botanical origins [1,2]. The large amounts of bioactive constituents endow bee pollen with health beneficial properties, such as antioxidant, anti-inflammatory, anti-allergen, anti-aging, and anti-cancer effects [3,4,5]. Owing to its nutritional and therapeutic properties, bee pollen has gained increasing attention worldwide and is commercially consumed as a natural dietary supplement for human health promotion [6,7].

Camellia bee pollen (CBP) is among the most important bee pollen products that are extensively consumed in China. It is gathered by honeybees from the flowers of tea plants (*Camellia sinensis* L.), the leaves of which can be made into tea, a popular beverage worldwide with various health benefits. It has been reported that the chemical constituents of tea flowers are similar to those of tea leaves [8]. A share of some common bioactive constituents and functional properties between CBP and tea can thus be expected. Indeed, CBP has a special fragrance, similar to the aroma of tea. Moreover, it has been demonstrated that CBP possesses higher anti-inflammatory, antioxidant, and anti-tyrosinase activities relative to other types of bee pollen [9,10,11]. In recent years, the identification of bioactive constituents responsible for the observed functional properties has been attracting growing interest. Among them, caffeine, kaempferol, levulinic acid, and 5-hydroxymethyl furfural are reported to contribute partly to the anti-tyrosinase activities of CBP [12,13,14]. However, the metabolic basis for its functional properties is still far from being fully understood, thereby impeding the use of CBP in the cosmetics, food, and pharmaceutical industries.

The aforementioned superior functional properties promote increasing demand for CBP, which leads to fraudulent practices in the market [15]. To identify bee pollen of different botanical origins, sensory testing (e.g., color, aroma, and taste characteristics) and microscopic examination (e.g., size, form, and color of pollen grains) are widely used [16]. However, such subjective judgments based on sensory evaluation are easily biased by personal preference. Moreover, even with a microscope, it is still difficult to distinguish between different types of bee pollen with similar morphological and structural attributes [17,18]. The situation is even worse for CBP, which shows substantial morphological variation between tea cultivars [19]. The lack of accurate identification methods represents a loophole for the current adulteration chaos of bee pollen. A more sensitive method is, thus, urgently needed to ensure accurate identification for the long-term development of the bee product industry.

Non-targeted metabolomics based on high-resolution mass spectrometry provides a convenient method for the simultaneous analysis of hundreds or thousands of small molecules in food products, including various bee products [20,21,22,23]. This approach, combined with targeted metabolomics, plays a key role in screening and quantifying marker compounds for food authenticity [24,25,26]. For bee pollen authenticity, however, such research is currently limited [27].

Our study aimed to uncover distinctive metabolic components of CBP and to explore efficient metabolite indicators to identify authentic CBP. To this end, non-targeted metabolic profiling of CBP and other types of bee pollen was performed. We proposed epicatechin gallate (ECG) and L-theanine as marker compounds of CBP, and measured their content based on ultra-high performance liquid chromatography-quadruple-Exactive Orbitrap mass spectrometry (UHPLC-Q-Exactive Orbitrap-MS).

## 2. Materials and Methods

### 2.1. Reagents and Standards

Ultrapure water was produced using a Milli-Q water purification system (Millipore, St. Louis, MA, USA). Methanol of LC-MS grade was purchased from Merck (Darmstadt, Germany). Acetonitrile, formic acid, and ammonium formate of LC–MS grade were purchased from Thermo Fisher Scientific (Waltham, MA, USA). All of the authentic standards used for qualification are listed in Table S1.

### 2.2. Bee Pollen Sample Collection

Fifteen CBP samples were collected from Anhui, Fujian, Jiangsu, Sichuan, and Zhejiang Provinces in China (*n* = 3 for each), while fifteen non-CBP samples were obtained from five botanical plants (*n* = 3 for each; Table S1), i.e., rose (*Rosa rugosa* Thunb.), apricot (*Prunus armeniaca* L.), lotus (*Nelumbo nucifera* Gaertn.), rape (*Brassica campestris* L.), and wuweizi (*Frucus Schisandra chinensis*). To guarantee their authenticity, these samples were collected by professional beekeepers from their apiaries of *Apis mellifera* L. colonies using pollen traps, and were then identified using a scanning electron microscope (S-4800, Hitachi, Tokyo, Japan). Dead bee parts and other hive debris were removed manually. All samples were freeze-dried and stored at −80 °C until analysis.

### 2.3. Preparation of Bee Pollen Extracts

In brief, 25 mL of 80% methanol was added to accurately weighed 0.5 g samples of powdered bee pollen in a 50 mL vial. After supersonic extraction for 1.5 h at 4 °C, the mixture was kept still for 30 min, followed by 0.22 μm membrane filtration (Shimadzu, Shanghai, China). Tolbutamide and sulfacetamide, at final concentrations of 2 μg/mL and 4 μg/mL, respectively, were added as internal standards for retention time correction.

### 2.4. UHPLC-QTOF/MS-Based Non-Targeted Metabolomics Analysis

Non-targeted metabolomics analysis was performed on an Infinity 1290 UHPLC system (Agilent Technologies, Santa Clara, CA, USA) coupled to an Agilent 6545 QTOF mass spectrometer (Agilent Technologies, Santa Clara, CA, USA). Chromatographic separation was carried out on a Zorbax Eclipse Plus C18 column (3.0 × 150 mm, 1.8 µm, Agilent Technologies, Santa Clara, CA, USA) at 40 °C. Water with 5 mmol/L ammonium acetate and methanol with 5 mmol/L ammonium acetate were used for mobile phases A and B, respectively, which were kept at a flow rate of 0.40 mL/min with a gradient elution profile. The proportion of solvent B was linearly applied as follows: 0–5 min, 0–12%; 5–15 min, 12–35%; 15–18 min, 35–45%; 18–26 min, 45–75%; 26–33 min, 75–95%; and 33–35 min, 95–5%. The injection volume was 3 μL. The post-time between each two consecutive injections was 3 min. Dual jet stream electrospray ionization (ESI) was performed under negative ionization mode. The parameters were as follows: nebulizer pressure: 35 psi; capillary voltage: 3.5 kV; gas flow rate: 8 L/min; fragmentator voltage: 130 V; sheath gas temperature: 350 °C; and sheath gas flow rate: 8 L/min. The TOF scan was set at an *m/z* of 100–1100 with an acquisition rate of 2 spectra per second. The auto MS/MS model was applied for compound identification with fixed collision energies (10 V, 20 V and 40 V). Reference ions with *m/z* 112.9856 and 1033.9881 were utilized for real-time mass calibration during both the TOF scan and the auto MS/MS scan.

The obtained raw data were imported into Masshunter Qualitative Analysis software (B.07.00 SP1, Agilent Technologies, Santa Clara, CA, USA) to extract all feature ions, then exported as .cef documents. These .cef documents were imported into MPP (Mass Profiler Professional software package, version B.14.5, Agilent Technologies, Santa Clara, CA, USA) for retention time correction using internal standards and subsequent peak alignment within the specified retention time window (±2.5%). The entities with an occurrence frequency >60% and a coefficient of variability (CV) < 25% were retained. After Pareto scaling and logarithmic transformation of the quantitative data, principal component analysis (PCA) was performed using SIMCA 14.1 (Umetrics AB, Umeå, Sweden) to provide an intuitionistic demonstration of an overall clustering pattern of the bee pollen samples.

Differential entities (*p* < 0.05 in analysis of variance, ANOVA) among the bee pollen samples from different botanical origins were identified using SPSS 20.0 (Chicago, IL, USA) for further analysis. Metabolite identification was performed by searching for exact mass and MS/MS spectra in the Metlin database (http://metlin.scripps.edu, accessed on 10 January 2021) and Human Metabolome Database (HMDB, https://hmdb.ca/, accessed on 10 January, 2021). The retention time and MS/MS spectra of putatively identified compounds were validated by authentic standards analyzed under the same conditions. To show the abundance differences in identified compounds among these bee pollen samples, heatmap visualization was carried out using MetaboAnalyst 4.0 [28] with Pareto scaling and logarithmic transformation. To improve the classification of CBP and non-CBP samples, orthogonal projections to latent structures discriminant analysis (OPLS-DA) was conducted in SIMCA 14.1. The OPLS-DA model was cross-validated by permutation tests with 200 iterations. The values of variable importance in projection (VIP) were used to rank the overall contribution of each compound to the OPLS-DA model. Compounds with VIP > 1.0, *p* < 0.05 according to Student’s *t*-test and fold change (FC) > 1.5 were regarded as discriminating compounds driving the observed group separation.

### 2.5. Targeted Quantification of ECG and L-Theanine in CBP

The contents of ECG and L-theanine in CBP were determined using the UHPLC system (Dionex Ultimate 3000, Thermo Scientific, Waltham, CA, USA) coupled with Q-Exactive Orbitrap-MS (Thermo Scientific, Waltham, CA, USA) in parallel reaction monitoring (PRM) mode. Compound separation was performed using hydrophilic interaction liquid chromatography (HILIC) with an ACQUITY BEH Amide column (150 mm × 2.1 mm, 1.7 μm, Waters, Milford, MA, USA) at 50 °C. Mobile phase A and B were 30% and 95% acetonitrile, respectively, both containing 10 mmol/L ammonium formate and 0.1% formic acid. A gradient elution profile at a flow rate of 0.3 mL/min was set as follows: 1 min, 100% B; 11 min, 30% B; 11.5 min, 100% B; and 15 min, 100% B. The injection volume was 2 μL. The Q-Exactive Orbitrap-MS with a heated HESI source was operated in negative mode with stepped normalized collision energies (NCE 10, 20, and 30). The mass resolution was 70,000 full width at half maximum (FWHM) for the full MS mode and 17,500 FWHM for the MS/MS scan. Calibration curves were established with standard solutions (0.5, 1.0, 2.0, 4.0, 6.0, 8.0, and 10.0 μg/mL for ECG and 5, 10, 20, 40, 60, 80, and 100 μg/mL for L-theanine) and used to calculate the ECG and L-theanine content in Xcalibur (v4.0.27.19, Thermo Fisher Scientific, Waltham, MA, USA).

## 3. Results

### 3.1. Overall Metabolic Profiles of the Bee Pollen

**Figure 1:**
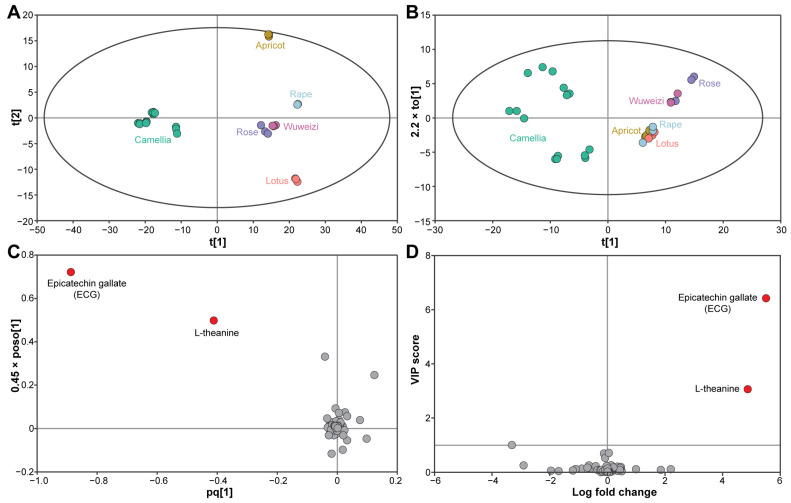
Multivariate and univariate analysis of CBP and non-CBP (rose, apricot, lotus, rape, and wuweizi bee pollen) metabolomics data. PCA score plots were generated from all valid entities (**A**); score plots (**B**) and loading plots (**C**) of OPLS-DA for the 54 identified compounds. The ellipses indicate 95% confidence limits according to Hotelling’s T2 statistics. VIP score and log fold change (CBP/non-CBP) of the identified compounds are shown (**D**).

To obtain an overview of grouping patterns of the bee pollen samples of different botanical origins, 670 valid entities were submitted with which to perform unsupervised PCA. The first two principal components of PCA explained 74.46% of the total variance (PC1 = 65.70% and PC2 = 8.76%). In the PCA score plots (Figure 1A), a tight clustering of the pollen samples from the same botanical origins was observed, and their distribution patterns were found to be affected by their botanical origins. Remarkably, a clear separation between the CBP and non-CBP samples was observed. Specifically, the CBP samples were all distributed in the negative part of the PC1 axis, while the non-CBP samples were located in the positive part of the PC1 axis and separated along the PC2 axis.

### 3.2. Metabolite Identification

**Figure 2:**
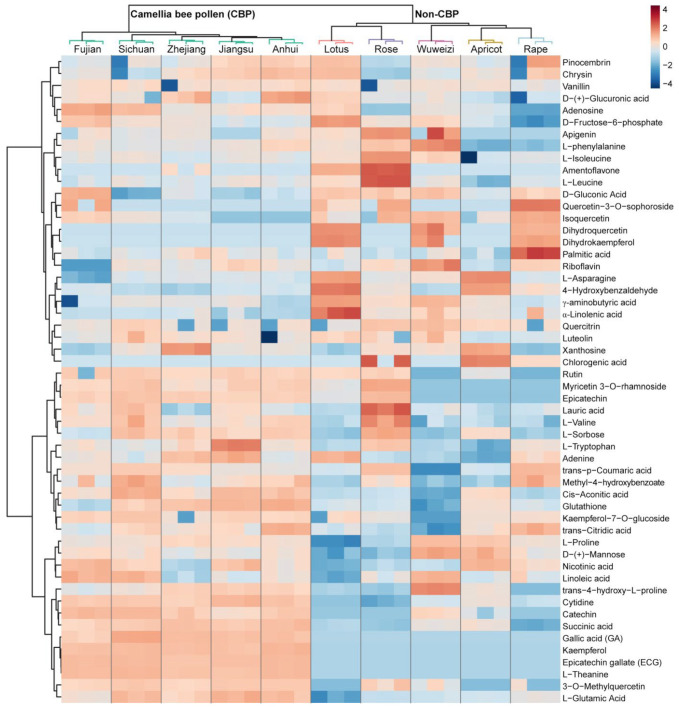
Clustering heatmap of the 54 identified compounds in the 30 bee pollen samples.

To screen the compounds explaining the overall grouping patterns observed in the PCA score plots, differential entities among the bee pollen samples (*p* < 0.05 in ANOVA) were subjected to compound identification. Finally, 54 compounds were identified, including 15 flavonol glycosides and flavone glycosides, 3 catechins, 11 amino acids, 8 organic acids, 4 fatty acids, 4 nucleotides and their derivatives, 2 aldehydes, and 7 other compounds (Table S1). Among them, four compounds were detected exclusively in all CBP samples, including ECG, L-theanine, gallic acid (GA), and kaempferol. The CBP samples were all clustered into a single clade, whereas the non-CBP samples formed a different clade in the clustering heatmap of the identified compounds (Figure 2).

### 3.3. Marker Compound Selection of CBP

To pick out the most discriminating compounds between the CBP and non-CBP samples, univariate and multivariate analyses were conducted based on the relative abundance levels of the 54 identified compounds (Table S2). In total, 16 compounds with FC > 1.5 showed a significant difference (*p* < 0.05) between the CBP and non-CBP samples (Table S1). A reliable OPLS-DA model was established (*R*^2^*Y* = 0.873, *Q*^2^ = 0.845, *R*^2^ intercepts = 0.0902, and *Q*^2^ intercepts = −0.4112 in a 200-time permutation test), and the resulting score plots supported a clear separation between the CBP and non-CBP samples (Figure 1B), as is consistent with the grouping patterns in the PCA score plots (Figure 1A). Further filtering with VIP values > 1.0 in the OPLS-DA resulted in a final selection of two compounds, i.e., ECG and L-theanine, which had the highest VIP values (Figure 1D). Moreover, ECG and L-theanine exhibited the greatest distance from the origin in the loading plots (Figure 1C) and, hence, had the highest discriminatory power. Taken together, ECG and L-theanine could be used as marker compounds to distinguish CBP from non-CBP samples.

### 3.4. ECG and L-Theanine Quantification

**Figure 3:**
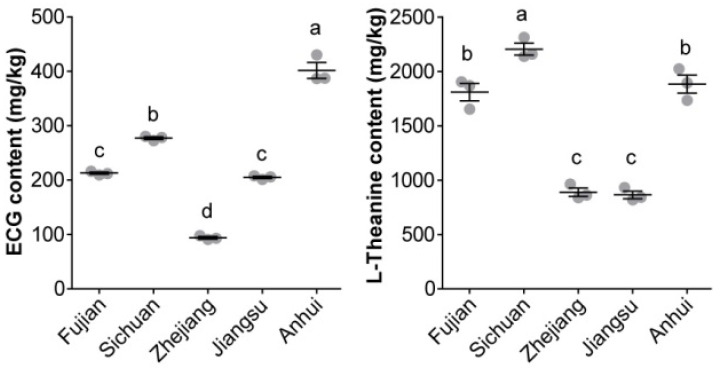
ECG and L-theanine content (mean ± standard error) in the CBP samples from different locations. Different letters indicate statistically significant differences (*p* < 0.05 in ANOVA).

A targeted quantification method based on a PRM assay was carried out to measure the ECG and L-theanine content in the CBP samples. The established calibration curve showed good linearity for ECG (*r*^2^ = 0.9965) and L-theanine (*r*^2^ = 0.9932). A significant difference was observed in the ECG (94.08–401.82 mg/kg) and L-theanine (864.83–2204.26 mg/kg) content among the CBP samples (Figure 3). The highest ECG content was found in the CBP samples from Anhui Province (387.04–430.70 mg/kg), followed by Sichuan Province (272.52–280.56 mg/kg), and the lowest content was found in the samples from Zhejiang Province (90.95–98.33 mg/kg). The CBP samples from Sichuan Province had the highest L-theanine content (2138.56–2314.01 mg/kg), while those from Jiangsu and Zhejiang Provinces had the lowest content (819.45–964.43 mg/kg).

## 4. Discussion

### 4.1. Distinctive Metabolic Profiles of CBP

As a mixture of flower pollen and honeybee saliva, bee pollen from different botanical origins shows differing chemical composition and functional properties [2,29]. Our metabolomics analysis confirmed the presence of different metabolic profiles of bee pollen samples according to botanical origins. Remarkably, one of our key findings was the distinctive metabolic profile of CBP samples compared with others. It has been reported that tea plants synthesize unique metabolites and transport quality-related components to their organs, including tea flowers [30], which could account for our observed special metabolic profile of CBP. Indeed, our study revealed four characteristic compounds (L-theanine, ECG, kaempferol, and GA) which were found to be specific to the CBP samples and 12 other differential compounds between CBP and non-CBP samples.

A wide diversity of bioactive functions has been reported for our identified differential compounds. Among them, L-theanine, a non-protein amino acid, is reported to naturally occur mainly in tea plants and shows a wide range of beneficial effects, such as antioxidant, anti-cancer, and immune-modulating activities [31,32]. ECG, a highly abundant catechin in green tea, has been demonstrated to possess antioxidant, anti-inflammatory, and anti-tumor effects [33]. Other polyphenolic compounds, such as kaempferol, GA, and rutin, show similar biological activities [34,35]. Collectively, these bioactive compounds account for at least some of the superior functional activities of CBP which have been reported in previous studies [9,10,11].

### 4.2. ECG and L-Theanine as Maker Compounds of CBP

Metabolomics approaches have been widely adopted for the global evaluation of marker compounds for food authenticity [24]. If some compounds are detected exclusively in certain bee products, or are significantly more abundant or profile-defining, they could be considered as markers of these products [36]. Typically, compounds with VIP scores greater than 1.0 in the OPLS-DA are generally considered to have the highest discrimination potential [37]. With these methods, marker compounds of bee products of different origins, such as honey [38] and propolis [23], have been proposed. In our study, ECG and L-theanine satisfied the conditions mentioned above (FC > 1.5, *p* < 0.05, and VIP score > 1.0), and could, thus, be regarded as the best potential candidates for CBP marker compounds. In addition, flavonoid glycosides, which are present in lower quantities in CBP, have been proposed to distinguish CBP from several kinds of bee pollen [17]. Unlike these flavonoid glycosides, ECG and L-theanine were found exclusively in CBP in our study. Based on the measured content in our study, a minimum content of 90.95 mg/kg for ECG and 819.45 mg/kg for L-theanine are required for the authentication of CBP. The combination of the two special components specific to CBP could, thus, assist in distinguishing CBP from adulterated CBP or other bee pollen.

It should be noted that significant variation in the content of both ECG (90.95–430.70 mg/kg) and L-theanine (819.45–2314.01 mg/kg) was observed in our CBP samples from different geographical locations. This finding could be explained by diverse tea germplasm resources and environmental conditions, which have been reported to affect L-theanine content in the young shoots of tea trees [39,40]. It is, thus, likely that the geographical origins of CBP could be predicted by means of ECG and L-theanine content after extensive sampling of CBP in future studies.

## 5. Conclusions

Our comparative metabolomics analysis revealed distinctive metabolic profiles of the CBP relative to other bee pollen, including rose, apricot, lotus, rape, and wuweizi bee pollen. Among the differential compounds, L-Theanine and ECG were detected exclusively in all the CBP samples, and showed the highest discriminatory power. Further quantification based on targeted metabolomics demonstrated the content of L-theanine (819.45–2314.01 mg/kg) and ECG (90.95–430.70 mg/kg) in the CBP samples. The feasibility of easy detection and quantification of ECG and L-theanine in bee pollen demonstrates their possible practical application as marker compounds for CBP authentication.

## Data Availability

Data is contained within the article.

## Supplementary Materials

The following supporting information can be downloaded at: https://www.mdpi.com/article/10.3390/foods12142661/s1, Table S1: Compounds identified in bee pollen samples. Table S2: Relative abundance of the identified compounds in bee pollen samples.
